# Small Extracellular Vesicles from Breast Cancer Cells Induce Cardiotoxicity

**DOI:** 10.3390/ijms26030945

**Published:** 2025-01-23

**Authors:** Jhon Jairo Osorio-Méndez, Luis Alberto Gómez-Grosso, Gladis Montoya-Ortiz, Susana Novoa-Herrán, Yohana Domínguez-Romero

**Affiliations:** 1 Master in Biochemistry Program, Department of Physiological Sciences, Faculty of Medicine, Universidad Nacional de Colombia, Bogotá 111321, Colombia; josoriome@unal.edu.co; 2 Molecular Physiology Group, Sub-Direction of Scientific and Technological Research, Direction of Public, Health Research, National Institute of Health, Bogotá 111321, Colombialydominguezr@unal.edu.co (Y.D.-R.); 3 Department of Physiological Sciences, Faculty of Medicine, Universidad Nacional de Colombia, Bogotá 111321, Colombia; 4 Doctorate in Biotechnology Program, Faculty of Sciences, Universidad Nacional de Colombia, Bogotá 111321, Colombia

**Keywords:** small extracellular vesicles (sEVs), breast cancer, cardiac cells, cytokines, cardiotoxicity, cardio-oncology, doxorubicin

## Abstract

Cardiovascular diseases and cancer are leading global causes of morbidity and mortality, necessitating advances in diagnosis and treatment. Doxorubicin (Doxo), a potent chemotherapy drug, causes long-term heart damage due to cardiotoxicity. Small extracellular vesicles (sEVs) carry bioactive molecules—such as proteins, lipids, and nucleic acids—that can modulate gene expression and signaling pathways in recipient cells, including cardiomyocytes. Through the delivery of cytokines, microRNAs, and growth factors, sEVs can influence cell survival, which plays a critical role in the development of cardiotoxicity. This study investigates the role of sEVs derived from breast cancer cells treated or not with Doxo and their potential to induce cardiomyocyte damage, thereby contributing to cardiotoxicity. We isolated sEVs from MCF-7 cells treated or not to Doxo using ultracentrifugation and characterized them through Nanoparticle Tracking Analysis (NTA), Scanning Electron Microscopy (SEM), and Western Blotting (WB) for the markers CD63, CD81, and TSG101. We analyzed cytokine profiles using a Multiplex Assay and Cytokine Membrane Array. We exposed Guinea pig cardiomyocytes to different concentrations of sEVs. We assessed their viability (MTT assay), shortening, reactive oxygen species (ROS–DHE dye) production, mitochondrial membrane potential (JC-1 dye), and calcium dynamics (FLUO-4 dye). We performed statistical analyses, including *t*-tests, ANOVA, Cohen’s d, and η^2^ to validate the robustness of the results. Treatment of MCF-7 cells with 0.01 μM Doxorubicin resulted in increased sEVs production, particularly after 48 h of exposure (~1.79 × 10^8^ ± 2.77 × 10^7^ vs. ~5.1 × 10^7^ ± 1.28 × 10^7^ particles/mL, *n* = 3, *p* = 0.0019). These sEVs exhibited protein profiles in the 130–25 kDa range and 93–123 nm sizes. They carried cytokines including TNF-α, IL-1β, IL-4, IFN-γ, and IL-10. Exposure of cardiomyocytes to sEVs (0.025 μg/mL to 2.5 μg/mL) from both Doxo-treated and untreated cells significantly reduced cardiomyocyte viability, shortened cell length by up to 20%, increased ROS production, and disrupted calcium homeostasis and mitochondrial membrane potential, indicating severe cellular stress and cardiotoxicity. These findings suggest that Doxo enhances sEVs production from breast cancer cells, which plays a key role in cardiotoxicity through their cytokine cargo. The study highlights the potential of these sEVs as biomarkers for early cardiotoxicity detection and as therapeutic targets to mitigate cardiovascular risks in chemotherapy patients. Future research should focus on understanding the mechanisms by which Doxorubicin-induced sEVs contribute to cardiotoxicity and exploring their diagnostic and therapeutic potential to improve patient safety and outcomes in cancer therapy.

## 1. Introduction

Patients with cancer may have a higher risk of developing cardiovascular diseases (CVDs), even in the absence of cancer treatment [1,2,3]. Common risk factors such as chronic inflammation, oxidative stress, and metabolic dysregulation contribute to the combined challenges of managing both cancer and CVD [4]. This dual burden complicates treatment and leads to increased rates of illness and death among patients [5]. Cardiotoxicity, a critical concern during cancer therapy, is mainly associated with chemotherapeutic agents like Doxo [6,7]. While Doxo’s efficacy in treating malignancies is well-established, its adverse effects on the cardiovascular system, especially in individuals with pre-existing CVD, are well-documented [8,9,10,11,12]. Recent evidence suggests that chemotherapy alone may not cause cardiotoxicity. Breast cancer cells can release small extracellular vesicles (sEVs) that cause cardiac damage independently of any treatment [13,14,15].

Extracellular vesicles are nanometer-sized structures crucial in intercellular communication by transporting bioactive molecules, including proteins, lipids, and nucleic acids [16,17,18,19,20]. Different types of cells secrete these vesicles, which can act locally or reach distant tissues, such as the heart, where they may influence the behavior of recipient cells like cardiomyocytes [21,22,23,24,25,26,27,28]. By delivering their molecular cargo, sEVs can modulate gene expression and activate signaling pathways in cardiomyocytes, potentially leading to cellular stress and damage [21,23,26,29]. Specifically, sEVs carry pro-inflammatory cytokines, such as TNF-α, IL-6, and IL-1β, which may induce inflammation, oxidative stress, and apoptosis in cardiac cells [28,30,31]. This inflammatory response disrupts cellular homeostasis and contributes to endothelial dysfunction, a key mechanism underlying the pathogenesis of CVDs [5,7].

In addition to inflammation, sEVs significantly influence mitochondrial dysfunction, which is a crucial factor in the development of cardiotoxicity. Mitochondria regulate energy production and cell survival in cardiomyocytes, and their dysfunction is a hallmark of both CVDs and cancer [32,33]. sEVs derived from breast cancer cells can disrupt mitochondrial dynamics, impair oxidative phosphorylation, and destabilize mitochondrial membrane potential, resulting in reduced ATP production and increased reactive oxygen species (ROS) generation [33,34]. Such mitochondrial impairments can activate apoptotic pathways, resulting in the death of cardiomyocytes. Importantly, this dysfunction may originate from signals carried by sEVs from untreated cancer cells, suggesting that cancer itself predisposes patients to cardiovascular complications, regardless of the treatment they receive of therapy [30].

sEVs pose a complex threat to cardiovascular health by integrating inflammation and mitochondrial dysfunction mechanisms. Pro-inflammatory cytokines cause systemic and localized vascular damage, while mitochondrial impairments disrupt the energy balance and survival of cardiomyocytes, increasing the risk of cardiotoxicity. Understanding the intersection of these mechanisms, particularly in the context of sEVs derived from both untreated and treated cancer cells, is essential to evaluate cardiac injury comprehensively [35,36].

In addition to their direct effects on cardiomyocytes, sEVs modulate the immune response, complicating their role in cardiotoxicity. Through their interactions with immune cells, sEVs can enhance inflammatory pathways or trigger protective responses, depending on their molecular cargo [30,37,38]. This dual role influences the tumor microenvironment, promoting angiogenesis, metastasis, and immune evasion while also affecting healthy tissues like the heart, thereby linking cancer progression to cardiovascular damage [30,39].

This study hypothesizes that sEVs derived from untreated and Doxo-treated breast cancer cells (MCF-7) contribute to cardiotoxicity by promoting mitochondrial dysfunction and impairing cardiomyocyte viability. The primary objectives were to isolate sEVs from MCF-7/breast cancer cells, characterize the physical, protein, and cytokine profiles of sEVs to identify key factors that may drive cardiotoxicity, and evaluate the effects of these sEVs on cardiomyocyte morphology, viability, intracellular calcium regulation, ROS production, and mitochondrial membrane potential. By uncovering the dual impact of sEVs from untreated and treated cancer cells, this research aims to provide critical insights into cardiovascular risks associated with cancer and its treatment, paving the way for innovative diagnostic and therapeutic strategies.

## 2. Results

The experimental design aimed to identify the optimal Doxo concentration and conditioning time for generating sEVs from MCF-7 cells while ensuring minimal impact on cell viability. We treated MCF-7 cells with different concentrations of Doxo and assessed cell viability at various incubation times to determine the most favorable conditions for sEVs isolation.

After isolating the sEVs, we characterized them biochemically and physically, focusing on particle concentration, size, exosome protein markers, and cytokines. We then applied these sEVs to isolated cardiomyocytes. We evaluated their effects on cell viability, oxidative stress levels, and intracellular calcium to investigate the potential cardiotoxic effects of sEVs derived from MCF-7 cells.

### 2.1. Optimal Doxorubicin Concentration for sEVs Isolation from MCF-7 Cancer Cells

**Figure 1:**
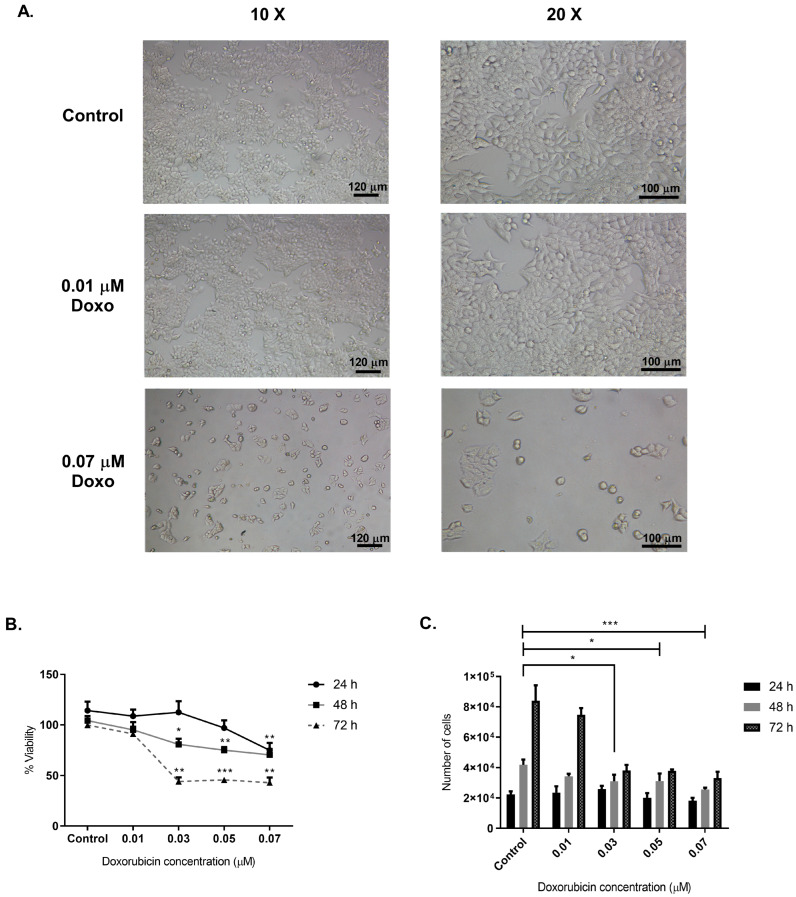
Determination of Doxo concentration to obtain conditioned media and subsequent isolation of sEVs. (**A**) Representative photographs of MCF-7 cells unexposed (Control) and exposed to Doxo (0.01 and 0.07 µM) at 10*X* and 20*X* magnifications at 48 h. (**B**) Evaluation of MCF-7 cell viability at 24-, 48-, and 72-h post-treatment (hpt) with different concentrations of Doxo (0, 0.01, 0.03, 0.05, and 0.07 µM). (**C**) Assessment of the effect of different concentrations of Doxo on the number of MCF-7 cells after 24, 48, and 72 hpt with different concentrations of Doxo. The viability estimations represent three replicates from three independent biological samples (three different cell cultures and treatments). We performed significance analysis of the assays using ANOVA with significance levels denoted as * for *p* < 0.05, ** for *p* < 0.01, and *** for *p* < 0.001. These results suggest that Doxo’s concentration of 0.001 µM is useful for obtaining a conditioned medium to isolate sEVs.

To determine the optimal concentration of Doxo and conditioning time for generating sEVs from MCF-7 cells, we treated the cells with Doxo at concentrations of 0, 0.01, 0.03, 0.05, and 0.07 µM, assessing cell viability at 24, 48, and 72 h (Figure 1A,B). Control cells showed stable viability across all time points. At 24 h, 0.07 µM Doxo significantly reduced viability by 34% (*p* = 0.0201) (Figure 1A). At 48 h, 0.03 µM Doxo caused a 22.5% reduction (*p* = 0.0248), with higher doses of 0.05 and 0.07 µM further decreasing viability by 28.08% and 32.52% (*p* = 0,0058 and *p* = 0.0083), respectively. By 72 h, 0.03 µM Doxo lowered viability by 55.81% (*p* = 0.0014), with 0.05 and 0.07 µM resulting in reductions of 54.18% and 56.9% (*p* = 0.006 and *p* = 0.0035) (Figure 1B).

After assessing cell viability, we evaluated the effect of Doxo treatment on cell numbers in culture. At 24 h (Figure 1C), we observed no significant differences between the treated and control groups. However, at 48 h, Doxo at 0.03 µM reduced cell numbers to 31,042 ± 4260, a decrease of approximately 25.87% compared to untreated cells (41,875 ± 3363, *p* = 0.0217). Similarly, 0.05 µM Doxo led to a 25.47% reduction (31,208 ± 4888, *p* = 0.0241), while 0.07 µM Doxo caused a 39% decrease in cell numbers (25,542 ± 1283, *p* = 0.0006). At 72 h, Doxo concentrations of 0.03, 0.05, and 0.07 µM reduced cell numbers to 38,125 ± 3683, 37,875 ± 901, and 33,125 ± 4131, respectively, corresponding to decreases of approximately 54.64%, 54.93%, and 60.59% compared to controls (84,042 ± 10,125, *p* < 0.0001) (Figure 1C). These findings indicate that 0.01 µM Doxo was the optimal concentration for generating conditioned media and isolating sEVs, as it preserved cell viability and maintained cell numbers. This concentration effectively balanced cell survival with the production of biologically relevant sEVs, minimizing apoptotic bodies’ presence and ensuring that the sEVs reflected the intended cellular response.

### 2.2. Isolation and Characterization of sEVs from Conditioned Media of Doxo-Treated MCF-7 Cells

**Figure 2:**
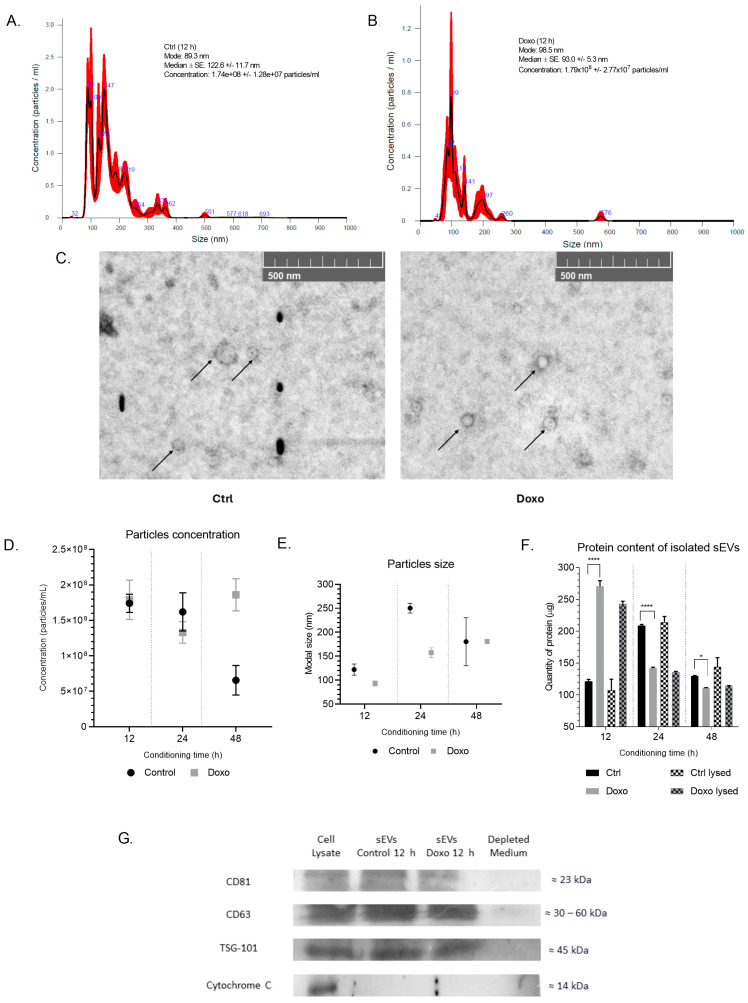
Physical and biochemical characterization of the sEVs population isolated from MCF-7 conditioned media. (**A**,**B**) Results of nanoparticle tracking analysis of sEVs isolated at 100,000× *g* from supernatants of MCF-7 cells treated or not with 0.01 µM Doxo for 48 h. The graph shows the mean (black line) and standard error of the mean from the five analyses (in red), along with the average concentration/size of FTLA per experiment. (**C**) SEM analysis showed a spheroid shape with a size of less than 200 nm (scale bar = 500 nm). The arrows show the sEVs. (**D**) Particle concentration at the three conditioning times (12, 24, and 48 h) by NTA analysis. (**E**) Particle size at each conditioning time (**F**) Quantification of protein content in intact sEV and lysed with 2% SDS and cycles of heating–cooling with liquid nitrogen. We performed significance analysis of the assays using ANOVA with significance levels denoted as * for *p* < 0.05 and **** for *p* < 0.0001. (**G**) Western blot analysis of sEVs markers (CD81, CD63, and TSG101) and control marker of exosomal origin (CYT C). The nanoparticle tracking analysis of isolated sEVs is representative of two different measurements. We evaluated the total cell extract from MCF-7 cells and the sEVs fraction from MCF-7 cells treated with DMSO (vehicle control) and Doxo for 12 h. Findings demonstrate successful isolation of sEVs with consistent size, shape, and protein markers across conditions, ensuring biological relevance.

To isolate sEVs, we performed ultracentrifugation on conditioned media collected from MCF-7 cells treated with 0.01 μM Doxo at various time points and from untreated cells. We concentrated the conditioned media using ultrafiltration and subsequent buffer exchange with PBS to eliminate potential contaminants from the DMEM culture medium, such as phenol red. We characterized the isolated vesicles using Nanoparticle Tracking Analysis (NTA), which revealed a heterogeneous population of particles with sizes ranging from 93 to 123 nm at the 12 h time point (Figure 2A,B). We further corroborated these results by electron microscopy, which confirmed that the isolated vesicles exhibited a uniform round shape with sizes between 50 and 150 nm (Figure 2C).

Interestingly, Doxo treatment led to a higher vesicle yield at 48 h, with the concentration of vesicles in Doxo-treated cells (1.86 × 10^8^ ± 2.27 × 10^7^ particles/mL) being significantly higher than in control cells (6.55 × 10^7^ ± 2.08 × 10^7^ particles/mL) (*p* = 0.0019, t = 7.27, df = 4). However, control cells and Doxo-treated cells generated similar concentrations of vesicles at 12 h (1.74 × 10^8^ ± 1.28 × 10^7^ particles/mL vs. 1.79 × 10^8^ ± 2.77 × 10^7^ particles/mL, respectively) (Figure 2D,E). Therefore, we chose to use the conditioned media and isolated vesicles from this time point for further biological assays.

We performed biochemical characterization of sEVs by SDS-PAGE electrophoresis on 30 µg of protein from each sample, and Coomassie Blue staining revealed a faint band at around 70 kDa in the sEVs fraction, along with two bands in the microvesicle (MV) fraction. Silver staining indicated that Doxo-treated sEVs were more protein-enriched than the total extract and MV fractions, with a protein range between 130 and 25 kDa. The MV fraction displayed a more conserved profile, with complete bands between 250 and 15 kDa. The depleted vesicle fraction showed a reduction in band numbers, retaining protein bands primarily in the 130 to 25 kDa range (Supplementary Figure S2). We categorized the protein content of sEVs into intact sEVs (surface proteins) and lysed sEVs (total proteins, including encapsulated and surface proteins). Doxo-treated MCF-7 cells showed peak secretion of sEVs at 12 h, while control cells peaked at 24 h. Detergent lysis showed a minimal increase in protein detection, suggesting that most proteins were located on the sEV surface (Figure 2F).

The analysis of sEVs protein content revealed changes over time due to Doxo treatment in MCF-7 cells. At 12 h, intact sEVs from Doxo-treated cells contained 270.42 ± 8.8 µg/mL, significantly higher than the 121.14 ± 3.12 µg/mL in controls (*p* < 0.0001). Lysed sEVs followed a similar trend, with Doxo-treated vesicles containing 243.04 ± 3.96 µg/mL compared to 107.25 ± 17.35 µg/mL in controls, a 126.62% increase (*p* < 0.0001).

At 24 h, Doxo-treated sEVs exhibited reduced protein levels compared to controls. Intact sEVs showed a 31.92% decrease (142.17 ± 1.30 µg/mL vs. 208.84 ± 1.72 µg/mL, *p* < 0.0001), while lysed sEVs displayed a 36.63% reduction (135.81 ± 1.08 µg/mL vs. 214.31 ± 8.68 µg/mL, *p* < 0.0001). By 48 h, protein levels equalized, though Doxo-treated sEVs remained slightly lower, with a 14.23% decrease in intact vesicles (111.01 ± 0.52 µg/mL vs. 129.43 ± 0.96 µg/mL, *p* = 0.0324) and a 20.57% reduction in lysed vesicles (114.40 ± 0.23 µg/mL vs. 144.02 ± 14.52 µg/mL, *p* = 0.020). These results highlight the impact of Doxo treatment on sEVs protein dynamics over time.

To confirm the presence of specific sEVs markers, we performed Western blotting on the total cell extract, the sEVs fractions from DMSO- and Doxo-treated cells at 12 h, and the vesicle-depleted medium. The analysis confirmed the presence of sEVs surface proteins in the enriched samples, such as CD81, TSG101, and CD63, validating successful sEVs isolation. Notably, the absence of Cytochrome C in the sEVs fraction confirmed that the isolated vesicles were free from mitochondrial contamination (Figure 2G).

These findings demonstrated that MCF-7 cells release small to medium-sized vesicles, with Doxo treatment increasing vesicle content at 12 h. The sEVs isolated from both Doxo-treated and control cells at 12 h exhibited similar size and concentration profiles, making this time point ideal for further biological assays to investigate the impact of Doxo-induced sEVs on cardiomyocytes.

### 2.3. Differential Cytokine Profile in Doxorubicin-Exposed MCF-7 sEVs

**Figure 3:**
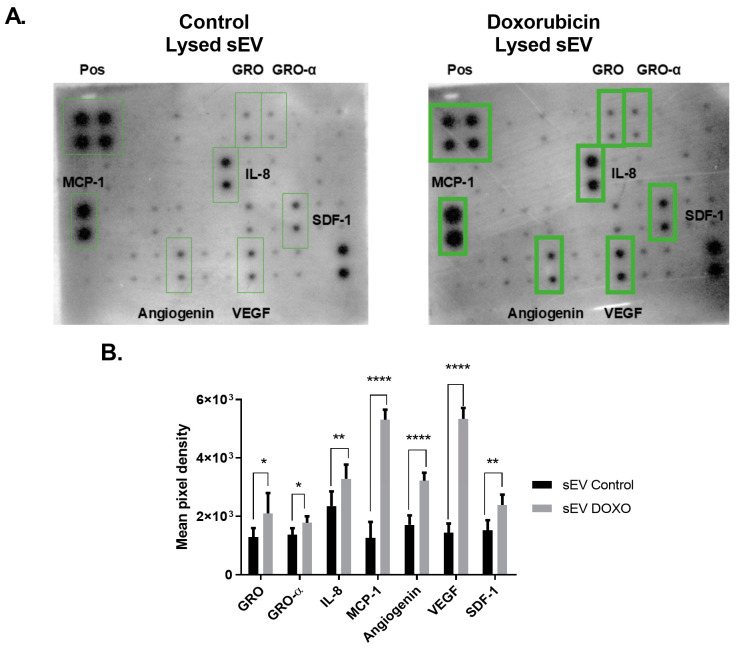
Cytokine profiling of 42 targets in sEVs from Doxo-treated and untreated MCF-7 cells. (**A**) Membrane cytokine arrays showing internal positive controls and highlighted cytokines (green boxes) with significant differential expression. (**B**) Mean pixel density analysis of differentially expressed cytokines. Data represent two independent experiments with biological duplicates (*n* = 2). Significance levels: * *p* < 0.05, ** *p* < 0.01, and **** *p* < 0.0001. These results suggest that Doxo enhances the pro-inflammatory cytokine content of sEVs, potentially contributing to cardiotoxicity.

sEVs carry signaling molecules, including miRNAs and cytokines, which can modulate cellular behavior and signaling pathways. Tumor-derived sEVs may mediate off-target effects, particularly those enriched with pro-inflammatory cytokines. To investigate the cytokine content of sEVs from MCF-7 cells exposed to Doxo, we utilized the Cytokine Array—Human Cytokine Antibody Array Kit (Membrane, 42 Targets). This analysis revealed the presence of a rich and diverse cytokine profile within these vesicles. Densitometric analysis of the membranes using ImageJ Fiji, software version !.54j revealed differential expressions of several cytokines. Analysis of lysed small extracellular vesicles (sEVs) from cells treated with Doxorubicin (Doxo) revealed significant increases in the levels of seven cytokines compared to control samples. Specifically, the relative optical densities (OD) of GRO increased by 63.5% (2100.56 ± 695.28 vs. 1285.17 ± 310; * *p* = 0.0108), and GRO-α rose by 29.8% (1775.76 ± 227.69 vs. 1368.17 ± 225.50; * *p* = 0.0349). IL-8 demonstrated a 39.7% increase (3284.07 ± 490.58 vs. 2351.47 ± 500.30; ** *p* = 0.0014), while SDF-1 levels increased by 63.5% (2489.01 ± 301.77 vs. 1524.62 ± 343.40; *** *p* = 0.0008) (Figure 3B).

MCP-1 exhibited the most substantial increase, rising by 387.5% (5309.73 ± 344.47 vs. 1089.26 ± 272.10; **** *p* < 0.0001). Additionally, angiogenin levels increased by 74.8% (3219.59 ± 274.47 vs. 1841.49 ± 109.78; **** *p* < 0.0001), and VEGF saw an increase of 247.2% (5239.64 ± 213.93 vs. 1509.18 ± 200.81; **** *p* < 0.0001) (Figure 3B).

These findings highlight the marked modulation of cytokines in sEVs derived from Doxo-treated cells, which may have implications for inflammatory and angiogenic signaling. The results suggest that Doxo treatment alters the cytokine content of sEVs, potentially affecting their ability to influence target cells compared to vesicles derived from untreated cells.

**Figure 4:**
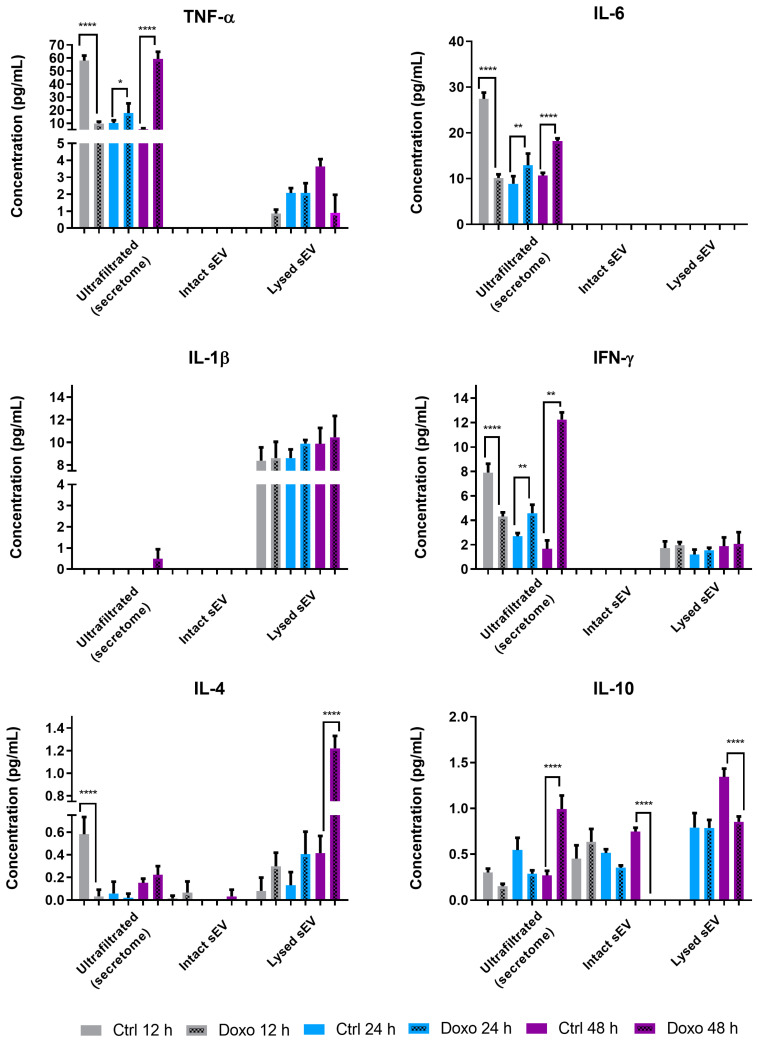
Concentration and distribution profiles of six cytokines (IFN-γ, IL-1β, IL-4, IL-6, IL-10, TNF-α) across soluble, lysed, and unlysed sEV fractions from Doxo-treated and untreated (Ctrl) MCF-7 cells. Statistical analysis via ANOVA; significance levels: * *p* < 0.05, ** *p* < 0.01, and **** *p* < 0.0001. Data include three technical replicates from three biological replicates per treatment condition (*n* = 3). These data highlight the role of cytokine compartmentalization in modulating sEVs-mediated effects on recipient cells.

Given the potential of cytokines such as IL-6, IL-1β, and TNF-α to induce inflammatory responses, we further explored whether Doxo treatment altered the cytokine composition in sEVs. We analyzed vesicle-associated and soluble cytokine fractions from ultrafiltered media using the MILLIPLEX MAP Human Cytokine Magnetic Bead Panel and the Human Cytokine Antibody Array kit. The ultrafiltration step, performed with Amicon Ultra 15 Merck Millipore devices, allowed us to distinguish cytokines localized on the vesicle surface, encapsulated within the vesicles, or released into the soluble fraction of the secretome (Figure 4).

We identified distinct cytokine distributions across compartments, with TNF-α predominantly localized in the soluble fraction (~90%) and a smaller fraction encapsulated within sEVs. TNF-α concentrations varied by conditioning time and treatment. At 12 h, UF Ctrl contained 58.02 ± 3.85 pg/mL, while UF Doxo significantly reduced to 9.79 ± 1.32 pg/mL (65.69% decrease, ****, *p* < 0.0001). At 48 h, UF Doxo showed a dramatic increase to 59.29 ± 5.47 pg/mL compared to 5.57 ± 0.58 pg/mL in UF Ctrl (963.8% increase, ***, *p* < 0.0001). TNF-α levels in lysed sEVs remained low and did not show significant differences between conditions or time points (e.g., sEVs Doxo 12 h: 0.87 ± 0.22 pg/mL) (Figure 4).

We detected IL-6 exclusively in the soluble fraction, with concentrations lower than TNF-α. At 12 h, UF Ctrl contained 27.45 ± 1.36 pg/mL, while UF Doxo decreased significantly to 10.14 ± 0.81 pg/mL (63.05% decrease, ****, *p* < 0.0001). At 48 h, UF Doxo levels increased to 18.20 ± 0.61 pg/mL, surpassing UF Ctrl levels (10.68 ± 0.58 pg/mL; 70.36% increase, ****, *p* < 0.0001). IL-1β was mainly encapsulated within sEVs, with concentrations ranging from 8.39 ± 1.18 pg/mL (sEVs Ctrl 12 h) to 10.44 ± 1.89 pg/mL (sEVs Doxo 48 h), but not significant differences emerged between treatments or conditioning times (Figure 4)

Approximately 60% of IFN-γ localized in the soluble fraction, and its secretion increased following Doxo exposure. At 48 h, UF Doxo reached 12.24 ± 0.58 pg/mL, a 633.34% increase compared to UF Ctrl (1.66 ± 0.68 pg/mL; ****, *p* < 0.0001). Encapsulation of IFN-γ within sEVs showed no significant differences, with concentrations ranging from 1.21 ± 0.39 pg/mL (sEVs Ctrl 24 h) to 2.06 ± 0.97 pg/mL (sEVs Doxo 48 h). IL-4 primarily localized in lysed sEVs, with the highest concentration (~1.22 ± 0.10 pg/mL) observed in Doxo-treated cells after 48 h, significantly higher than in untreated cells (~0.42 ± 0.15 pg/mL) (Figure 4).

Finally, IL-10 showed a heterogeneous distribution across soluble, lysed, and intact sEV fractions. Lysed sEVs contained the highest levels after 48 h of conditioning, with sEVs Ctrl at 1.34 ± 0.09 pg/mL compared to 0.80 ± 0.05 pg/mL in sEVs Doxo (75.27% decrease, ****, *p* < 0.0001). These findings demonstrate that Doxo treatment induces time-dependent and cytokine-specific alterations in soluble and vesicular fractions (Figure 4).

These findings highlight the intricate cytokine profile of sEVs derived from untreated and Doxo-treated MCF-7 cells, providing insights into their potential roles in intercellular communication and the modulation of inflammatory signaling pathways (Figure 3 and Figure 4).

### 2.4. Effect of MCF-7-Derived sEVs on Cardiomyocyte Viability, Cellular Morphology, Oxidative Stress, Intracellular Calcium Levels, and Mitochondrial Membrane Potential in Isolated Guinea Pig Cardiomyocytes

**Figure 5:**
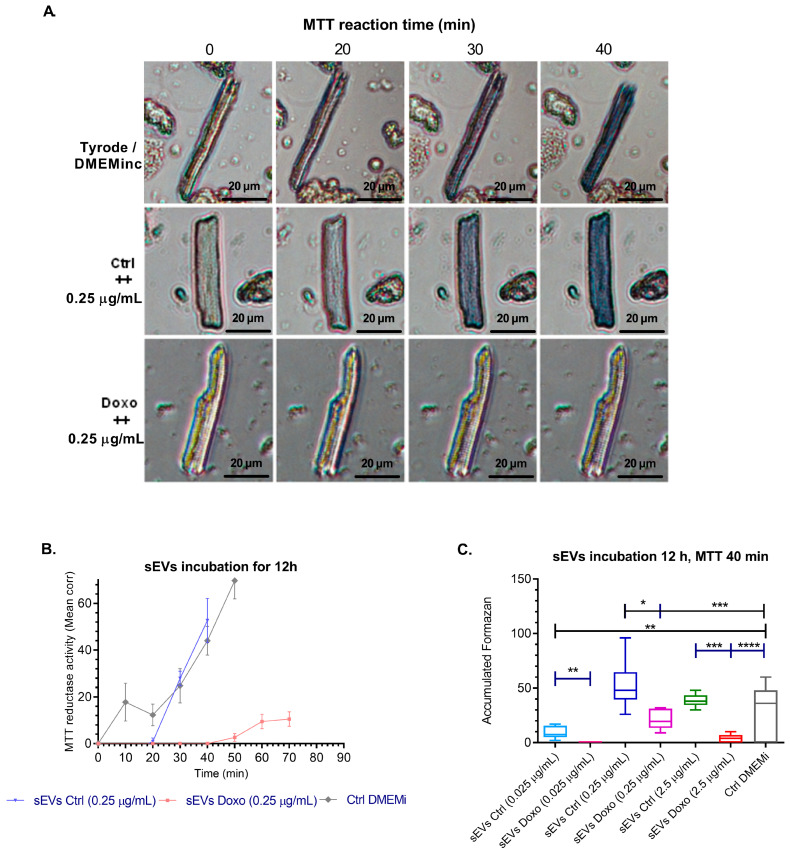
MTT reductase activity in isolated Guinea pig cardiomyocytes treated with different concentrations of vesicular proteins. This figure shows the corrected mean of formazan produced by cardiomyocytes incubated for 12 h with extracellular vesicles (sEVs) (100,000× *g*) derived from MCF-7 cells treated with 0.01 µM Doxo or the corresponding DMSO vehicle control. We evaluated the biological effects of sEVs using three different concentrations of vesicular protein: 0.025, 0.25, and 2.5 µg/mL, all dispersed in DMEM medium. The study exposed the control group of cardiomyocytes to DMEM medium only. (**A**) Formazan production results for cardiomyocytes treated with sEVs from control (0.25 µg/mL) and Doxo-treated (0.25 µg/mL) MCF-7 cells. (**B**) The figure shows the formazan production kinetics for each experimental group. (**C**) Statistical analysis was performed at 0, 50, and 70 min after MTT addition using a two-tailed unpaired Student’s *t*-test (*n* = 6). Significance levels were indicated as * *p* < 0.05, ** *p* < 0.01, *** *p* < 0.001, **** *p* < 0.0001. Results indicate that sEVs from Doxo-treated cells significantly impair cardiomyocyte metabolic activity compared to control sEVs.

Isolation of cardiomyocytes: Adult Guinea pig cardiomyocytes were successfully isolated and characterized for baseline viability and morphological characteristics. Viability assays, including the Trypan Blue exclusion test and MTT reductase activity measurements (Figure 5A,B), confirmed the integrity and metabolic activity of the isolated cells. We used bright-field microscopy imaging to assess cellular morphology, revealing that rod-shaped cardiomyocytes exhibited the highest mitochondrial activity, as indicated by intracellular formazan production (mean absorbance: 1.010 ± 0.012 OD). In contrast, populations dominated by round-shaped cells showed significantly lower metabolic activity (mean absorbance: 0.005 ± 0.001 OD) [40,41]. These findings validated the sensitivity and reliability of the MTT assay for detecting viability changes in response to experimental conditions.

We incubated cardiomyocytes with sEVs from MCF-7 cells treated with doxorubicin (0.01 µM, for 1, 12, and 24 h) or untreated cells. sEVs were assessed at three concentrations (0.025, 0.25, and 2.5 µg/mL) in DMEM medium, with control groups including vesicle-depleted conditioned medium, unfractionated conditioned medium, incomplete DMEM without fetal bovine serum (DMEMi), and Tyrode buffer. We exposed cardiomyocytes with sEVs derived from untreated or Doxo-treated MCF-7 cells to evaluate cardiomyocyte viability. We assessed metabolic activity using the MTT assay and determined membrane integrity through the Trypan Blue exclusion assay.

Metabolic changes were observed in cardiomyocytes exposed to sEVs derived from untreated MCF-7 cells, showing a significant increase in MTT reductase activity compared to control media. Treatment with sEVs from untreated cells (Ctrl, 0.25 µg/mL) induced formazan production at 30 min (27.84% ± 7.62), peaking at 40 min (52.6% ± 4.2). In contrast, cardiomyocytes treated with sEVs derived from doxorubicin-treated cells (Doxo, 0.25 µg/mL) began producing formazan at 50 min (2.67% ± 2.28) and reached their maximum at 70 min (12.6% ± 6.23). Untreated cardiomyocytes, serving as a baseline, initiated formazan production at 10 min (17.75% ± 9.90) and reached their peak at 50 min (69.75% ± 15.65) (Figure 5B). These findings indicate that doxorubicin treatment alters the cargo of sEVs, reducing their ability to support cardiomyocyte metabolism.

The kinetics and accumulated formazan production for each experimental group indicated that sEVs from untreated MCF-7 cells at 0.25 µg/mL induced faster and greater formazan production, indicating significantly higher MTT reductase activity than sEVs from Doxo-treated cells at the same concentration performed at 0, 50, and 70 min after MTT addition (Figure 5A–C). These findings suggest that sEVs from untreated MCF-7 cells stimulate cardiomyocyte metabolic activity compared to sEVs from Doxo-treated cells, based on the production of formazan and the shorter time in which formazan production begins, reducing metabolic activity. This could indicate that Doxo treatment alters the vesicular content and negatively influences cardiomyocyte function (Figure 5A–C).

**Figure 6:**
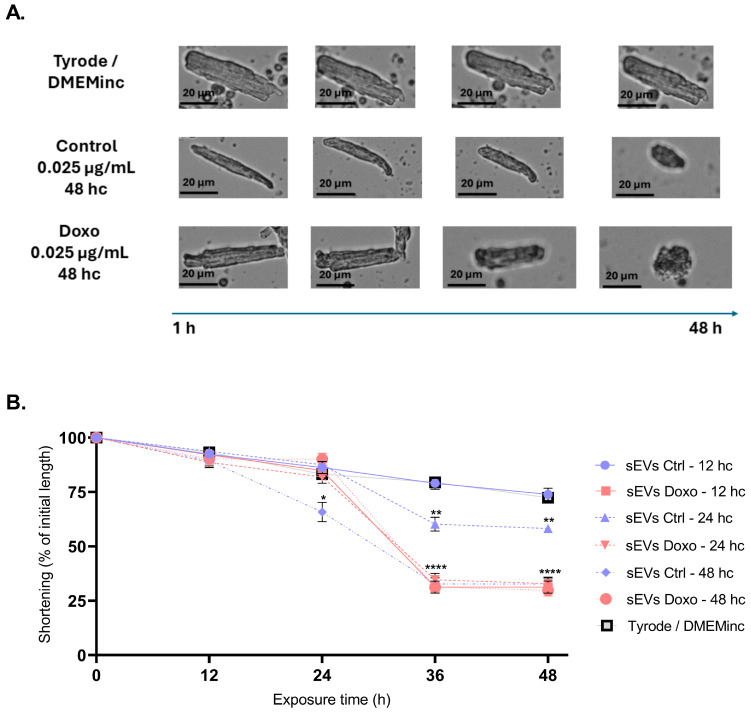
Effect of different concentrations of sEVs on the length of isolated Guinea pig cardiomyocytes. (**A**) A representative image shows cardiomyocyte shortening and loss of rod-like shape after treatment with 0.025 µg/mL concentration of sEVs from media conditioned for 12, 24, and 48 h. (**B**) Percentage of cardiomyocyte shortening treatment with a 0.025 µg/mL concentration of sEVs of three different conditioning times (12, 24, and 48 hc). Results represent three independent experiments using cardiomyocytes isolated from three different preparations and treated with sEVs (*n* = 15 per experiment). We performed statistical analysis using ANOVA for shortening after 48 h of treatment. Significance levels are indicated as * *p* < 0.05, ** *p* < 0.01 and **** *p* < 0.0001. Findings demonstrate that sEVs induce significant cardiomyocyte shortening, correlating with cellular stress and reduced viability.

Cardiomyocyte morphology analysis revealed significant reductions in cell size after treatment with sEVs: Cardiomyocytes treated with sEVs from 12 h conditioned media at a concentration of 0.025 µg/mL exhibited dramatic size reduction to 31.33 ± 8.43 µm after 36 and 48 h of treatment, consistent with a shortening of approximately 60.6% compared to the control (**** *p* < 0.0001). When we treated cardiomyocytes with sEVs from 48 h conditioned media, both 0.025 µg/mL sEV Ctrl 48 h and 0.25 µg/mL sEV Doxo 48 h induced severe reductions in cardiomyocyte size to 32.70 ± 9.06 µm (Figure 6B) and 29.78 ± 7.77 µm (Supplementary Figure S3A), respectively (**** *p* < 0.0001), reflecting an ~80% shortening compared to the control (72.43 ± 5.49 µm) (Figure 6A,B). These findings confirm that untreated and Doxo-treated MCF-7-derived sEVs induce substantial cardiomyocyte shortening, potentially correlating with reduced cell viability.

Evaluation of oxidative stress: The effects of sEVs from different conditioning times (12, 24, and 48 h) on ROS levels in isolated cardiomyocytes were influenced by the protein concentration and whether the vesicles were derived from Doxo-treated cells.

For sEVs from 12 h conditioned media, Doxo-enriched sEVs at 0.025 μg/mL moderately increased ROS levels in cardiomyocytes (η^2^ = 0.35; *p* = 0.0177), whereas a higher concentration (2.5 μg/mL) caused a significant rise in ROS (η^2^ = 0.73; *p* < 0.0001) (Supplementary Figure S3B). For 24 h conditioned sEVs, control vesicles at 0.025 μg/mL strongly increased ROS production (η^2^ = 0.73; *p* = 0.0002). Interestingly, sEVs derived from Doxo-treated cells at the same concentration also significantly increased ROS, though the effect was smaller (η^2^ = 0.59; *p* = 0.0009) (Supplementary Figure S3B).

**Figure 7:**
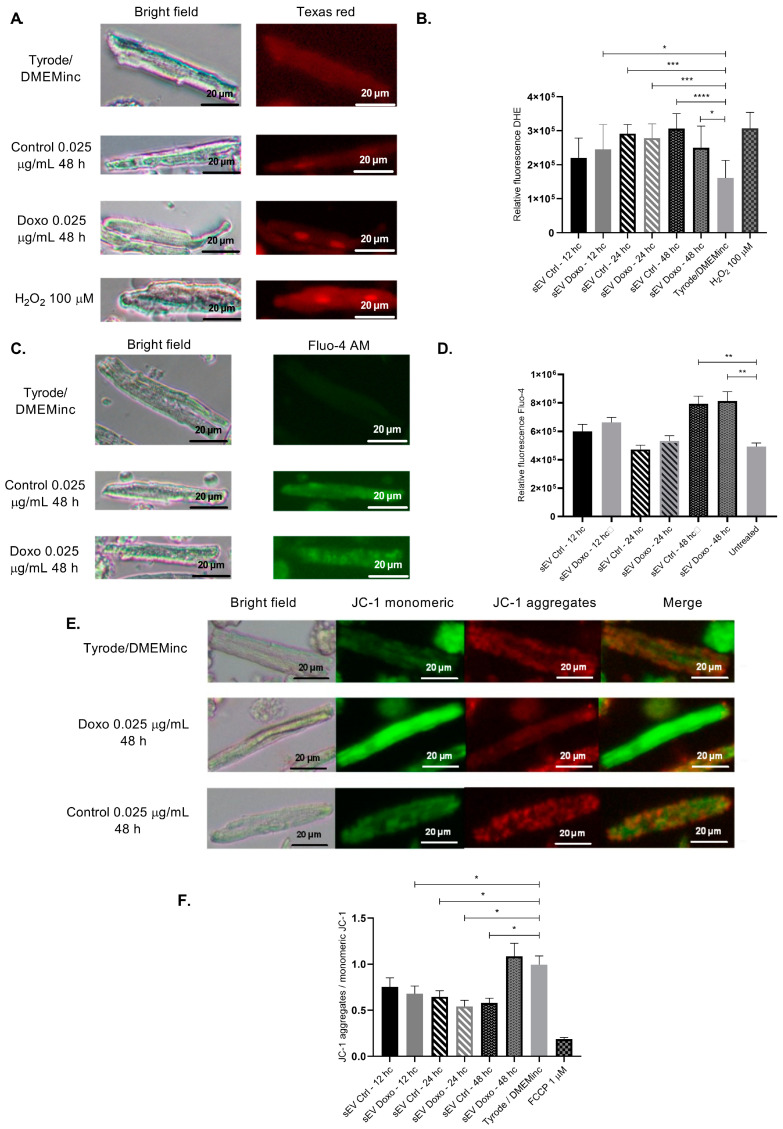
Effect of sEVs from three different conditioning times (12, 24, and 48 h of conditioning (hc)) on ROS (**A**,**B**), intracellular calcium (**C**,**D**), and mitochondrial membrane potential (**E**,**F**) in isolated Guinea pig cardiomyocytes. (**A**) Representative fluorescence microscopy images of cardiomyocytes in a bright field and fluorescence at approximately 585 to 590 nm. (**B**) Assessment of ROS production in isolated cardiomyocytes treated with 0.025 μg/mL of sEVs for 24 h. (**C**) Representative fluorescence microscopy images showing cardiomyocytes in a bright field and fluorescence at 514 to 529 nm. (**D**) Measurement of intracellular calcium levels in cardiomyocytes treated with 0.025 μg/mL of sEVs for 24 h. (**E**) Representative fluorescence microscopy images of cardiomyocytes in a bright field, with fluorescence at 514 to 529 nm and 585 to 590 nm. (**F**) Evaluation of mitochondrial membrane potential in cardiomyocytes treated with 0.025 μg/mL of sEVs for 24 h. ANOVA statistical analysis was performed on the mean fluorescence values for 15 cardiomyocytes per group (*n* = 15). The analysis used three measurements from three different isolations and treatments. Significance levels are indicated as * *p* < 0.05, ** *p* < 0.01, *** *p* < 0.001, **** *p* < 0.0001. We used the Shapiro–Wilk and Kolmogorov–Smirnov tests to assess normal data distribution and applied Bartlett’s test to assess equality of variance. ANOVA or ANOVA/Brown–Forsythe followed Bartlett’s test. Tukey’s or Tamhane’s T2 test was used for multiple comparisons. Effect sizes were determined using eta squared (η^2^) and Cohen’s d tests. These results underline the role of sEVs in inducing cardiomyocyte dysfunction through oxidative stress, calcium imbalance, and mitochondrial depolarization.

For 48 h conditioned sEVs, control vesicles at 0.025 μg/mL continued to show a significant effect on ROS levels (η^2^ = 0.72; *p* < 0.0001), whereas the Doxo-enriched sEVs showed a more moderate effect (η^2^ = 0.3; *p* = 0.0105). We observed the most pronounced effects on ROS production with vesicles derived from 24 h conditioned media (Figure 7A,B).

Evaluation of intracellular calcium handling: Intracellular calcium levels were measured using the calcium indicator Fluo-4 AM. The effects of sEVs from 48 h conditioned media on intracellular calcium levels in Doxo-treated cardiomyocytes varied depending on the concentration. At 0.025 μg/mL, control sEVs induced a moderate but non-significant increase in calcium levels (η^2^ = 0.58; *p* = 0.056). In contrast, Doxo-enriched sEVs at the same concentration produced a significant, moderate effect (η^2^ = 0.55; Cohen’s d = 0.38; *p* = 0.0160) (Figure 7C,D). Finally, at 2.5 μg/mL, control sEVs caused a significant and pronounced increase in calcium levels (η^2^ = 0.03; Cohen’s d = 2.32; *p* = 0.0016), showing an apparent deviation from the effects observed at lower concentration (Supplementary Figure S3D).

Evaluation of mitochondrial membrane potential (ΔΨm): We also evaluated the effects of control and doxorubicin-treated sEVs on mitochondrial membrane potential. Treatment with 12 h conditioned sEVs at 0.025 μg/mL of Doxo sEVs showed a moderate reduction in mitochondrial membrane potential (η^2^ = 0.25; Cohen’s d = 0.5; *p* = 0.0156). For sEVs from 24 h conditioned media, the control sEVs at 0.025 μg/mL had a moderate effect (η^2^ = 0.32; *p* = 0.0206), whereas Doxo sEVs at the same concentration had a more substantial impact on mitochondrial potential (η^2^ = 0.48; *p* = 0.0259) (Figure 7E,F).

At a higher concentration (2.5 μg/mL), both control (η^2^ = 0.62; *p* = 0.0049) and Doxo sEVs (η^2^ = 0.47; *p* = 0.0279) caused a pronounced disruption in mitochondrial membrane potential (Supplementary Figure S3B). Similar effects were observed with sEVs from 48 h conditioned media, where both control (η^2^ = 0.43; *p* = 0.0377) and Doxo sEVs (η^2^ = 0.43; *p* = 0.0346) significantly impaired mitochondrial potential. These findings highlight the strong impact of sEVs, particularly at higher concentrations, on mitochondrial integrity.

Integrating data from cellular viability, morphology, oxidative stress, calcium handling, and mitochondrial membrane potential assessments reveals a concentration-dependent impairment of cardiomyocyte function induced mainly by sEVs from Doxo-treated MCF-7 cells. This suggests that Doxo treatment may alter the vesicular cargo, negatively affecting cardiomyocyte function. The observed dysfunction includes increased oxidative stress, disrupted calcium homeostasis, compromised mitochondrial integrity, and metabolic impairment. Collectively, these findings provide strong evidence of the potential cardiotoxicity of MCF-7-derived sEVs in the context of doxorubicin treatment, underscoring the impact of doxorubicin treatment on vesicular content and its implications for cardiac health and the intrinsic deleterious nature of sEVs from MCF-7 cancer cells.

## 3. Discussion

Doxorubicin (Doxo)-induced cytotoxicity and cardiotoxicity are well-established phenomena attributed to mechanisms such as reactive oxygen species (ROS) production, topoisomerase IIβ-mediated DNA damage, and autophagy dysregulation [31,42]. However, the influence of small extracellular vesicles (sEVs) derived from Doxo-treated cancer cells on cardiac cells, especially ventricular cardiomyocytes, remains insufficiently characterized. This study addresses this gap by investigating the effects of MCF-7 cell-derived sEVs on ventricular cardiomyocytes, enhancing our understanding of their role in Doxo-induced cardiotoxicity and their potential as biomarkers for cardiovascular complications associated with cancer therapy.

### 3.1. Optimizing Doxorubicin Concentration to Induce Cytotoxic Stress in MCF-7 Cells for sEV Isolation and Functional Analysis

Our findings confirm that doxorubicin (Doxo) induces significant cytotoxic stress in MCF-7 cells in a dose- and time-dependent manner. Morphological alterations observed under microscopy, including cell shrinkage and detachment, strongly suggest apoptosis as a primary mechanism of cytotoxicity, consistent with Doxo’s established role as a DNA-damaging agent [42,43,44,45]. These results justify Doxo concentrations ranging from 0.01 µM to 0.07 µM to induce controlled cellular stress and generate conditioned media suitable for small extracellular vesicle (sEVs) isolation.

Quantitative analysis revealed that concentrations above 0.03 µM significantly reduced cell viability and proliferation, particularly at 48 and 72 h post-treatment. These findings corroborate previous studies highlighting the pro-apoptotic effects of Doxo in cancer cells and validate our decision to select 0.01 µM as the optimal concentration for subsequent experiments [46,47,48]. This dose effectively induces cytotoxic stress while minimizing excessive cell death, enabling the production of biologically relevant Doxo-modified sEVs for downstream functional analysis. Importantly, this approach balances cellular viability with sEVs yield, ensuring the relevance of the resulting vesicles in functional assays.

The evaluation of MCF-7 cell viability across a range of Doxo concentrations (0, 0.01, 0.03, 0.05, and 0.07 µM) at 24, 48, and 72 h post-treatment revealed a dose- and time-dependent decline in both cell viability and cell count (Figure 1B,C). Notably, concentrations above 0.03 µM elicited pronounced reductions in viability and proliferation, particularly at the 48 and 72 h time points. These reductions were statistically significant (ANOVA; *p* < 0.05, *p* < 0.01, *p* < 0.001, and *p* < 0.0001) and reinforced the choice of 0.01 µM as a concentration that induces stress without complete cytotoxicity. By optimizing this parameter, we ensured the generation of sEVs reflective of the Doxo-induced stress environment while preserving sufficient cell viability for vesicle secretion.

These findings confirm the effective concentration ranges of Doxo for generating conditioned media from MCF-7 cells and establish a critical foundation for investigating the biological effects of Doxo-treated sEVs on recipient cells, such as cardiomyocytes. The production of sEVs under controlled chemotherapeutic stress provides a unique opportunity to study their impact on cellular viability, stress responses, and toxicity in recipient cells. Moreover, this study underscores the significance of understanding the dynamics of sEVs secretion under chemotherapeutic stress, particularly in vesicle biogenesis and function. The observed changes in sEVs release and potential functional alterations in vesicle subtypes may shed light on mechanisms such as drug resistance, cellular survival, and the propagation of apoptotic signals [49].

This work provides valuable insights into the relationship between Doxo treatment, cellular stress, and sEVs secretion, offering a platform to explore further how these vesicles mediate intercellular communication and contribute to the systemic effects of chemotherapy. Future studies will aim to characterize the molecular cargo of Doxo-modified sEVs and assess their impact on cardiomyocytes, focusing on their role in cardiotoxicity and the modulation of pro-inflammatory and apoptotic pathways.

### 3.2. Effective sEVs Isolation and Characterization from MCF-7 Cells

Isolating and characterizing sEVs from MCF-7 cells treated with or without Doxo provide valuable insights into their structural stability and potential applications in functional studies. Nanoparticle tracking analysis (NTA) revealed that particle concentrations are maintained for conditioning times of 12, 24, and 48 h (Figure 2A,B), indicating that prolonged incubation enhances sEVs yield without compromising particle quality. The average particle size remained consistently below 200 nm, a characteristic feature of sEVs. Scanning electron microscopy (SEM) images confirmed this observation, showing uniform spheroid particles under 200 nm diameter (Figure 2C). These results reinforce the classification of these vesicles as small sEVs and suggest that optimizing conditioning duration can maximize vesicle production [50,51,52].

We observed comparable size and concentration distributions in sEVs from both Doxo-treated cells (0.01 µM) and untreated cells. This finding indicates that low-dose Doxo does not significantly alter the physical properties of the vesicles. The stability of these characteristics ensures that any observed biological effects on recipient cells will likely arise from changes in sEVs molecular content rather than alterations in vesicle structure or morphology [50,53,54,55].

Protein quantification demonstrated that intact sEVs maintained a stable protein load and successfully released protein after lysis with sodium dodecyl sulfate (SDS) and freeze–thaw cycles, indicating their structural integrity. Interestingly, protein levels decreased at 24 and 48 h (Figure 2), which may result from the reabsorption of sEVs by cancer cells, as previously reported by Matsumoto et al. (2017) [56]. Western blot analysis confirmed the presence of canonical exosome markers, including CD81, CD63, and TSG101 (Figure 2G), validating the vesicular origin of the isolates. Notably, the absence of cytochrome c (CYT C) excluded the possibility of cellular contamination, underscoring the high quality of the sEVs preparations.

These results confirm the successful isolation of a stable and homogenous sEVs population from MCF-7 cells, which retains its physical properties even after exposure to low-dose Doxo [13,57]. This stability is a pivotal factor for downstream functional studies, as it enables the attribution of biological effects of Doxo-treated sEVs directly to their molecular content rather than structural variations. Such high-quality characterization sets a robust foundation for investigating the role of Doxo-treated sEVs in mediating intercellular communication and their potential implications in drug resistance, cytotoxicity, and cellular stress responses in recipient cells.

### 3.3. Cytokine Profile of sEVs from Doxorubicin-Treated MCF-7 Cells

Small extracellular vesicles (sEVs) carry diverse bioactive molecules, including nucleic acids, proteins, and lipids, which can profoundly affect target cells. Our study highlights the cytokine profile of sEVs derived from doxorubicin (Doxo)-treated MCF-7 cells and its potential role in cardiotoxicity. Cytokines are pivotal mediators in acute and chronic heart failure, prompting us to investigate their differential distribution within sEVs from Doxo-treated cells. Notably, our results revealed distinct cytokine profiles (Figure 3 and Figure 4) with significant differences between the soluble cytokine fraction and those associated with intact or lysed sEVs, consistent with earlier studies [58,59,60].

Our findings demonstrated that Doxo treatment of MCF-7 cells induced a predominantly pro-inflammatory cytokine profile in sEVs, characterized by elevated levels of IL-6, TNF-α, IL-8, and IFN-γ (Figure 3). These results corroborate reports showing increased TNF-α production in Doxo-treated MCF-7 cells [11,30,61,62]. Furthermore, elevated IL-6, IL-8, and IL-1β levels in the MCF-7 secretome align with other studies linking these cytokines to inflammation and cardiac dysfunction [63].

The mechanisms guiding extracellular vesicle (sEVs) targeting remain poorly understood, leaving uncertainty about whether exosome delivery is stochastic or destination-specific. Once an exosome reaches its target cell, it can interact with plasma membrane receptors through surface proteins, fuse with the membrane, or undergo endocytosis via various pathways, including phagocytosis and clathrin- or caveolin-mediated mechanisms [56,64,65]. Surface molecules like tetraspanins, immunoglobulins, proteoglycans, and lectin receptors facilitate exosome binding, though the specifics remain unknown. Exosomal ligands such as IL-6, TNF-α, IL-8, and IFN-γ, and other molecules cargo are of cardiovascular interest, as their receptors on cardiac cells and vascular endothelial enable entry and potential reprogramming of target cells, influencing endothelial and cardiac functions. Notably, TNF-α and IFN-γ induce mitochondrial dysfunction, leading to nitric and oxidative stress in cardiomyocytes [66].

Our data also support the hypothesis of differential cytokine compartmentalization within sEVs. As reported for monocyte-derived vesicles, vesicles encapsulate specific cytokines (e.g., IFN-γ, IL-1β, IL-4, IL-10, TNF-α), while others, such as IL-6, predominate in the soluble fraction [59,60,67]. This selective packaging underscores the complex roles of sEVs in mediating cellular stress responses.

The analysis of lysed sEVs from Doxo-treated MCF-7 cells revealed increased levels of cytokines and chemokines, including GRO, VEGF, IL-8, MCP-1, angiogenin, and SDF-1. These molecules contribute to cardiomyocyte injury and inflammation. For example, although researchers do not fully understand the effects of GRO on cardiomyocytes, its pro-inflammatory nature suggests it impairs contractility, similar to TNF-α. Researchers have linked elevated IL-8 levels to myocardial infarction lesions, and MCP-1 plays a role in cardiac cell death and dysfunction [60,68,69]. These findings align with previous studies showing IL-8-induced mitochondrial fission and ROS production and MCP-1-mediated apoptosis and endoplasmic reticulum stress in cardiac cells [68,70].

VEGF, a key angiogenic factor, was also upregulated in Doxo-sEVs. Although VEGF can protect cardiomyocytes from ischemia-reperfusion injury, its association with Doxo treatment raises concerns about increased cardiovascular risk [7,71,72,73]. Similarly, SDF-1, implicated in ischemic heart disease and heart failure, may exacerbate TNF-α-induced cardiomyocyte death and dysfunction [73,74,75].

### 3.4. Cardiotoxic Effects of sEVs from MCF-7 Cells on Isolated Cardiomyocytes

This study explored the cardiotoxic effects of sEVs derived from MCF-7 cells, untreated and treated with Doxo, on isolated cardiomyocytes from female Guinea pigs. A key methodological decision was standardizing treatments based on protein content rather than sEVs concentration. Given the heterogeneity of sEVs preparations and the potential for protein aggregation, this approach reduced variability due to differences in purity and yield, aligning with best practices in extracellular vesicle research [50,51]. This normalization strategy enhances the reliability and biological relevance of the experimental findings.

We evaluated the viability of cardiomyocytes by measuring mitochondrial activity with MTT reductase and assessing cell morphology. Rod-shaped cells, which preserve structural integrity and mitochondrial function, exhibited the highest metabolic activity and viability. Conversely, hypercontracted or rounded cells showed diminished reductase activity, reflecting impaired mitochondrial function and reduced metabolic capacity. These findings emphasize cell morphology’s and mitochondrial health’s interdependence in maintaining cardiomyocyte viability under physiological and stress conditions. The sensitivity of the MTT assay, coupled with complementary methods like Trypan blue exclusion, enabled a comprehensive assessment of cardiomyocyte health, consistent with previous studies such as Gomez et al. (1997) [40].

Exposing cardiomyocytes to sEVs derived from untreated and Doxo-treated MCF-7 cells revealed a pronounced impact on mitochondrial function. Reduced MTT reductase activity indicated impaired mitochondrial metabolism, with sEVs from Doxo-treated cells having the most severe effects. Surprisingly, even sEVs from untreated MCF-7 cells adversely affected mitochondrial activity, suggesting that cancer cells inherently secrete vesicles carrying bioactive molecules capable of disrupting cardiomyocyte metabolism. This observation aligns with studies reporting that cancer cell-derived vesicles can mediate cellular damage independent of external treatments, underscoring the importance of examining the basal secretory profiles of cancer cells [76,77].

The cardiotoxicity of sEVs was further evident in the pronounced reduction in cardiomyocyte length and the increase in reactive oxygen species (ROS) production after 24 h of exposure (Figure 7). We also observed mitochondrial membrane depolarization and calcium dysregulation, critical markers of cellular stress and damage (Figure 7). These results are consistent with established mechanisms of Doxo-induced cardiotoxicity [6,7,11,63,78]. Rigorous ultracentrifugation and characterization protocols confirmed the absence of detectable residual Doxo in the sEVs preparations, ensuring that the observed effects were attributable to the vesicles, not residual drug contamination.

Furthermore, the sEVs from untreated MCF-7 cells also caused notable effects on cardiomyocytes, particularly after prolonged exposure (24–48 h). These findings reinforce the hypothesis that cancer cell-derived vesicles inherently carry factors that induce cellular stress. Similar observations have been reported by Cho et al., who demonstrated that exosomes from ovarian cancer cells induced a myofibroblastic phenotype in mesenchymal cells [74], and by Strnadová et al. and Yang et al., who reported that cancer cell-derived exosomes could promote inflammation, inhibit apoptosis, and activate survival pathways in non-cancerous cells [16,70].

Cytokine receptors on cardiomyocytes, such as those for IL-1 and TNF-α, suggest direct responsiveness to cytokines encapsulated in sEVs [30,39,66]. However, acknowledging that the observed cardiomyocyte phenotype results from the combined effects of cytokines and other bioactive molecules within sEVs, including miRNAs, lipids, and proteins, is essential. This complexity requires further research to dissect the molecular mechanisms by which sEVs contribute to cardiomyocyte dysfunction.

The results highlight the significant cardiotoxic potential of sEVs derived from untreated and Doxo-treated MCF-7 cells. The findings suggest ROS production, mitochondrial dysfunction, and calcium dysregulation mediated the adverse effects on cardiomyocytes. The inherent properties of cancer cell-derived vesicles, coupled with the modifications induced by Doxo treatment, underscore the need for further research to elucidate the molecular mechanisms underlying these effects, including determination of the cellular signaling pathways activated by sEVs-associated cytokines. In addition, future studies should focus on identifying the specific bioactive molecules within sEVs responsible for these cardiotoxic outcomes. Proteomic and genomic analyses could identify other molecules contributing to cardiotoxicity, while sEVs cytokine profiles hold promise as non-invasive diagnostic biomarkers for cardiovascular disease.

Equally important is the exploration of therapeutic strategies to mitigate their impact on cardiac health in oncology patients. In this context, it is also essential to highlight that age and gender are critical determinants in understanding cardiotoxic effects, particularly in the framework of personalized medicine [79,80,81]. Age-related physiological changes, such as declining liver and kidney function, can alter drug metabolism and clearance, leading to prolonged exposure and increased toxicity, as seen with agents such as doxorubicin [82,83,84,85]. Furthermore, aging is associated with structural and functional cardiovascular changes, including reduced cardiac output, myocardial thickening, and alterations in vascular tone, which increase susceptibility to cardiac injury [86,87,88]. The presence of comorbidities, such as hypertension or diabetes, further exacerbates these risks in older populations [3].

Gender differences also play a critical role. Estrogen confers cardioprotective effects in premenopausal women, an advantage that decreases after menopause and increases their susceptibility to cardiotoxicity [89,90]. Furthermore, gender differences in drug metabolism and pharmacokinetics influence the severity of toxic effects [91,92,93,94]. For example, a smaller heart size may result in higher local concentrations of cardiotoxic agents than in men. Recognition of these variables underscores the need for personalized approaches.

Adjusting drug doses, incorporating hormone-based therapies when appropriate, and implementing sex- and age-specific monitoring protocols can reduce cardiotoxic risks [82,95,96,97]. Furthermore, understanding these differences can guide the development of new cardioprotective strategies and safer therapeutic agents, ultimately improving patient outcomes.

In conclusion, while this study was conducted in vitro and significant gaps remain to be addressed, it lays a foundational framework for translational research, particularly in cancer patients undergoing Doxo therapy.

Study limitations, challenges/future focus

We should consider several limitations. First, we did not establish a causal relationship between Doxo-induced sEVs formation and their cardiotoxic effects. The characterization of sEVs confirmed the absence of residual Doxo, but researchers need to conduct further studies to understand the dynamics of sEVs internalization in cardiomyocytes. This study also highlights the role of sEVs in cardiotoxicity, but it has limitations. The reliance on in vitro models limits clinical relevance; future in vivo studies using transgenic models in mice or Guinea pigs are essential to validate the findings and assess the long-term effects of Doxo [98,99,100]. Similarly, the use of Guinea pig cardiomyocytes as human surrogates limits translational accuracy; *human induced pluripotent stem cell-derived cardiomyocytes* (hiPSC-CMs) should be prioritized to better model human responses and interindividual variability [6,101,102,103].

Another limitation is that short-term exposures (12–48 h) leave chronic effects unexplored; in this regard, longitudinal studies evaluating chronic exposure to sEVs with advanced imaging techniques such as live cell microscopy and intravital imaging are needed to assess persistent effects on cardiac tissue [104,105,106]. In addition, deeper molecular characterization of the sEVs cargo using multi-omics approaches such as proteomics, lipidomics, and transcriptomics may reveal novel therapeutic targets and pathways to mitigate chemotherapy-induced cardiotoxicity [98,107,108].

Despite these limitations, our study contributes valuable insights into the cardiotoxic potential of cancer cell-derived sEVs, emphasizing the importance of investigating their role in oncology-related cardiac complications.

Clinical perspectives

Early detection of cardiotoxicity can be improved using sensitive biomarkers such as sEVs, which carry various molecular materials reflecting cell health [109,110]. Monitoring these changes can identify patients at risk earlier, allowing for timely interventions that may enhance outcomes [109,111,112]. Tracking sEVs levels during treatments like chemotherapy can help assess disease progression and adjust therapies to minimize heart damage. Understanding the molecular content of sEVs can reveal mechanisms of cardiotoxicity. This insight might lead to new treatment targets and strategies, such as modifying specific sEVs to reduce toxic signals or delivering protective agents directly to the heart [110,113,114,115].

sEVs can be used for non-invasive diagnostics, as they can be collected from fluids like blood, urine, or saliva [109,114]. This method aligns with liquid biopsy trends in medicine. Additionally, sEVs can help personalize treatment plans based on the patient’s specific molecular profile, improving therapy responses while lowering side effects, and potentially allowing for integration into routine clinical practices [113,116,117].

## 4. Materials and Methods

### 4.1. Cell Culture and Doxorubicin Treatment

We cultured human breast cancer MCF-7 cells (ATCC^®^ HTB-22, American Type Culture Collection, Manassas, VA, USA) (ATCC^®^ HTB-22) in DMEM medium (Gibco—12100046, Thermo Fisher Scientific, Waltham, MA, USA) supplemented with 10% fetal bovine serum (FBS—Gibco—10270106, Thermo Fisher Scientific, Waltham, MA, USA) and penicillin/streptomycin at pH 7.4. We seeded the cells at 2 × 10^6^ in 175 cm^2^ culture flasks and maintained them at 37 °C with 5% CO_2_. After adherence, we treated the cells with Doxorubicin hydrochloride (Sigma Aldrich—D1515, St. Louis, MO, USA) at concentrations of 0.01, 0.03, 0.05, and 0.07 μM in DMSO (Thermo Scientific—D12345, Waltham, MA, USA) for 48 h. We assessed cell viability at specific Doxo concentrations (0.01, 0.03, 0.05, and 0.07 µM) using the Tali™ Image-Based Cytometer (Thermo Fisher Scientific, Waltham, MA, USA), Tali™ Viability Kit (Thermo Fisher Scientific, Waltham, MA, USA), Tali™ Apoptosis kit (Thermo Fisher Scientific, Waltham, MA, USA)and the Trypan Blue Exclusion assay [118] (*n* = 3; three different cell cultures, *p* ≤ 0.05).

### 4.2. Isolation of Extracellular Vesicles and Characterization Using Nanoparticle Tracking Analysis (NTA) and Western Blot

We isolated small extracellular vesicles (sEVs) from MCF-7 cells at 80% confluence by washing them with PBS and transferring them to serum-free DMEM after 48 h of Doxo treatment. The medium was conditioned for 12, 24, or 48 h and then clarified by centrifugation at 300× *g* for 10 min and 4500× *g* for 20 min and filtration. sEVs were purified by differential ultracentrifugation with minor modifications [26,66,68].

Equivalent volumes of culture medium (100 mL per 175 cm^2^ flask) were processed at constant cell density (1 × 106 cells/mL). After initial centrifugation at 300× *g* for 10 min and 4500× *g* for 20 min, the supernatant was filtered through a 0.22 µm Steriflip^®^—GP filter (MilliporeSigma, Burlington, MA, USA) and stored at –80 °C. The concentration was adjusted by ultrafiltration using an Amicon model 8200 stirred cell (MilliporeSigma, Burlington, MA, USA) and a Millipore Amicon^®^ Ultra-15 filter (10 kDa MWCO). The medium was centrifuged sequentially at 10,000× *g* and 19,000× *g* to remove larger vesicles, followed by ultracentrifugation at 100,000× *g* for 2 h at 4 °C. The pellet was resuspended in PBS, stored at –80 °C, and washed with 1X PBS.

We performed Nanoparticle Tracking Analysis (NTA) using the NanoSight NS300 system (Malvern Instruments Ltd., Malvern, Worcestershire, UK) to measure sEVs size distribution [119,120,121,122]. For Western blot, we quantified the exosome pool using the MicroBCA™ Protein Assay Kit (23235, Thermo Fisher Scientific, Waltham, MA, USA). The electrophoretic profile of the obtained sEVs was evaluated by separating 30 µg of protein from each sample via SDS-PAGE on a 12% polyacrylamide gel, followed by transfer to membranes. Blots were probed with antibodies against CD63 (TS63, ab59479, Abcam, Cambridge, UK), CD81 (M38, ab79559, Abcam, Cambridge, UK), TSG101 (4A10, ab83, Abcam, Cambridge, UK), and Cytochrome C (7H8, sc-13560, Santa Cruz Biotech, Dallas, TX, USA) and developed using an ECL Prime Western Blotting Detection Kit (Amersham, Little Chalfont, Buckinghamshire, UK) [16,69].

### 4.3. Scanning Transmission Electron Microscopy (STEM)

We performed negative staining by placing a sample drop on a copper grid and incubating it for 15 min [123,124]. Then, we removed the excess sample from the edge of the grid with filter paper. Next, we exposed the grid to 3% uranyl acetate, removed the excess with filter paper, and allowed it to dry. Finally, we examined the prepared samples using scanning transmission electron microscopy (STEM). Vesicle analysis was performed with a transmitted electron detector (R-STEM) attached to a scanning electron microscope (SEM Tescan model Lyra 3 Tescan Orsay Holding a.s., headquartered in Brno, South Moravian Region, Czech Republic) operating at an accelerating voltage of 30 kV.

### 4.4. sEVs Lysis and Cytokine Profiling

We added SDS to the exosomal fraction to assess the protein concentration in both lysed and intact sEV fractions to achieve a final concentration of 2% (*w*/*v*). Samples were subjected to three cycles of liquid nitrogen freezing and thawing at 100 °C, alternating with vortexing [55,56]. We added protease and phosphatase inhibitors to prevent sample degradation. We measured protein concentration using the MicroBCA™ Protein Assay Kit ((Thermo Fisher Scientific, Waltham, MA, USA), using a Bovine Serum Albumin (BSA) standard curve, and verified it by immobilizing proteins onto a nitrocellulose membrane [37,125]. Next, we stained the membrane with 0.1% (*w*/*v*) Amido black in 45% methanol and 10% acetic acid.

We analyzed the cytokine content of sEVs using a Cytokine Human Membrane Antibody Array (ab133997, Abcam, Cambridge, United Kingdom) to measure 42 target cytokines [32,126,127,128]. We used approximately 500 µg of protein from fractions obtained from media conditioned for 12 h from cells treated with Doxo. We treated the small vesicle fraction with 2% SDS and applied freeze-thaw cycles in liquid nitrogen to lyse the vesicles and evaluate surface and encapsulated cytokines. Following the manufacturer’s instructions, we performed the assays and obtained both fractions’ cytokine profiles. In addition, we analyzed six cytokines, namely, IFN-γ, IL-1β, IL-4, IL-6, IL-10, and TNF-α, with the MULTIPLEX MAP Human Cytokine Magnetic Bead Panel—Immunology Multiplex Assay kit (MilliporeSigma, Burlington, MA, USA) and the samples were normalized to the lowest concentration among all samples [58,70,71].

### 4.5. Cardiomyocyte Isolation, Vesicle Treatment, and Imaging Analysis

We chose isolated Guinea pig cardiomyocytes as a study model because they represent a valuable model for assessing biological effects, as their cardiac physiology is remarkably similar to that of humans [129,130,131]. Likewise, this model allows for a more detailed study of interactions in an environment that better reflects the complexity of cardiac tissue compared to more simplified cellular models, such as immortalized cell lines [132].

As described in previous protocols, ventricular cardiomyocytes were isolated by enzymatic dissociation [40,41,132]. We anesthetized adult Guinea pigs (200–300 g) with pentobarbital (12 mg/100 g body weight, intraperitoneally). After performing a cardiotomy, we retrogradely perfused the heart at 37 °C with Medium 199 (Sigma, St. Louis, MO, USA) for 2–3 min to remove blood. This was followed by perfusion with a Ca^2+^-free EGTA-buffered “low-Ca^2+^ medium” (calcium potential 7) for 180–240 s, containing (in mM): NaCl 100, KCl 10, KH_2_PO_4_ 1.2, MgSO_4_ 5, glucose 20, taurine 50, HEPES 10 (pH 7.2–7.4). The heart was then perfused with a low-Ca^2+^ medium containing pronase E (0.8%, Sigma), proteinase K (1.7%, Promega, Madison WI, USA), bovine albumin (0.1%, fraction V, Sigma), and 200 mM CaCl_2_. We separated the ventricles from the atria and cut them into tiny fragments (6–10 mm^3^) in the low-Ca^2+^ medium.

We isolated single cardiomyocytes by shaking the tissue at 37 °C in the same solution supplemented with collagenase (0.025 mg/mL, Sigma). After 20 min, we filtered the cell suspension through a nylon mesh, centrifuged it at 350× *g*, and washed it twice with the low-Ca^2+^ solution. We subjected the remaining tissue fragments to additional collagenase exposure for up to three cycles to ensure optimal cell yield. We obtained enriched populations of rod-shaped ventricular myocytes through differential centrifugation and decantation, eliminating round cells and debris. We stored the isolated cardiomyocytes in Tyrode’s solution containing (in mM): Glucose 5.5, NaCl 136.5, KCl 5.4, MgCl_2_ 0.53, HEPES 5.5, and CaCl_2_ 1.8. We visualized and imaged the cells using a Nikon inverted microscope and captured images with a Nikon Digital Sight DG U3 camera. The Institutional Committee at the National Institute of Health of Colombia approved all experimental protocols for using and caring for animals.

We used an MTT assay to assess the effect of sEVs derived from MCF-7 human breast cancer cells, with and without Doxo exposure, on cardiomyocyte viability. The assay followed protocols for adult ventricular cardiomyocytes with minor modifications [40,41].

We monitored cell morphology and the development of purple formazan color in real-time for 45–120 min, which provided data for subsequent morphometric and colorimetric analyses. We quantified formazan production by dissolving the product in 15% (*v*/*v*) DMSO and measuring absorbance at 540 nm using a Beckman DU64 spectrophotometer. We performed the experiments at room temperature (21 ± 2 °C), using MTT reductase activity as a viability indicator, where viable cells showed activity and non-viable cells showed no activity [30]. Unless otherwise stated, we used analytical grade reagents for cardiomyocyte culture, which we purchased from Sigma (St. Louis, MO, USA). We prepared solutions as stock concentrations and diluted them immediately before use. We assessed reactive oxygen species (ROS) production in cardiomyocytes using the DHE assay kit (ab236206, Abcam, Cambridge, United Kingdom) and fluorescence microscopy equipped with a Texas red filter, which allowed us to monitor ROS dynamics.

To evaluate the effects of different concentrations of sEVs from MCF-7 cells treated with Doxo on cardiomyocytes, we treated approximately 1 × 10^4^ cardiomyocytes with sEVs at protein concentrations of 0.025, 0.25, or 2.5 µg/mL, diluted in incomplete DMEM medium, for 1, 12, or 24 h. Control groups included sEVs from MCF-7 cells treated with vehicle control (DMSO), conditioned medium (unfractionated), EVs-depleted medium, incomplete DMEM medium as the sEVs vehicle, and Tyrode buffer.

We performed brightfield imaging of treated cardiomyocytes using a Cytation™ 3 high-content imaging system (BioTek, Winooski, VT, USA) with a 10× objective. We analyzed ventricular cardiomyocyte length and percent shortening by measuring the ratio of final length (FL) to initial length (IL) during the exposure times. We processed images using Fiji/ImageJ software (v 1.53q, Wayne Rasband, NIH, Bethesda, MD, USA) Wayne Rasband, NIH, USA) and calculated the percentage of shortening based on changes in cell length [41,132].

### 4.6. ROS Levels Assessment in Cardiomyocytes with DHE Dye

We quantified Reactive Oxygen species (ROS) levels in cardiomyocytes according to the manufacturer’s protocol for the Dihydroethidium (DHE) Assay Kit—Reactive Oxygen Species (ab236206) [132,133]. Fluorescence microscopy was performed using a Nikon Eclipse Ni-E fluorescence microscope (Nikon Corporation, Tokyo, Japan) equipped with a Texas Red filter (AT560/40/610 LP) at 20× magnification, and images were captured with a Nikon Digital Sight DS-U3 camera (Nikon Corporation, Tokyo, Japan). We measured red fluorescence intensity as an indicator of ROS production using image analysis techniques similar to those described previously.

### 4.7. Mitochondrial Membrane Potential Assessment in Cardiomyocytes with JC-1 Dye

Mitochondrial membrane potential in cardiomyocytes was assessed using JC-1 Dye (Invitrogen T3168, Eugene, OR, USA), following the manufacturer’s protocols. Cardiomyocytes were treated as described previously [27,134,135]. Imaging was conducted with a Nikon Eclipse Ni-E fluorescence microscope with a Nikon Digital Sight DS-U3 camera at 20× magnification. We identified polarized mitochondria by their punctate orange-red fluorescence, while depolarized mitochondria showed diffuse green monomer fluorescence, according to the manufacturer’s specifications.

### 4.8. Evaluation of Intracellular Calcium Levels by Fluo-4 AM Reagent

Following the manufacturer’s instructions, we measured intracellular calcium levels in cardiomyocytes using Fluo-4 AM, a cell-permeant calcium indicator dye (Invitrogen F14201). Cardiomyocytes were treated as previously described [132,136]. Fluorescence images were captured using a Nikon Eclipse Ni-E fluorescence microscope equipped with a Nikon Digital Sight DS-U3 camera at 20× magnification. A bright green fluorescence signal was used to indicate the presence of intracellular calcium upon Ca^2+^ binding.

### 4.9. Statistical Analysis

We present data as mean ± SEM, with *n* representing the number of experiments. We performed statistical analyses using GraphPad Prism 8.0 (GraphPad Software, Northampton, MA, USA) (Corp. Northampton, MA, USA). We used Student’s *t*-test (paired or unpaired, as appropriate) to assess differences between two means and one-way analysis of variance (ANOVA) for comparisons between more than two groups. Because cardiomyocyte isolation from a single Guinea pig typically yields 2 to 3 million cells, allowing for multiple experiments, our investigations focused on cell-level outcomes rather than between-subject comparisons. We designed the study using a within-subject approach, utilizing the same population of cardiomyocytes for both experimental and control conditions to reduce variability and ensure robust control.

For the MTT assay, calcium measurements, and mitochondrial membrane potential experiments, comparisons were made using a two-tailed unpaired Student’s *t*-test, with the same cardiomyocyte population serving as the control (*n* = 6–15 cells per assay; 95% CI, from two biological replicates of guinea pigs).

The statistical analysis process included several steps: Researchers performed the Shapiro–Wilk and Kolmogorov–Smirnov tests to assess the normality of data distribution thoroughly. Homogeneity of variances: We carefully selected and used Bartlett’s test to evaluate group equality. We used ANOVA or the ANOVA/Brown–Forsythe test to analyze group differences, depending on the results. Post hoc Comparisons: Where appropriate, post hoc tests such as Tukey’s or Tamhane’s T2 were used to compare multiple groups. Effect size: We computed effect size using Eta squared (η^2^) and Cohen’s. ANOVA was chosen for its robustness in handling multiple group comparisons. Additionally, we elaborated on the calculation of effect sizes, including Cohen’s d and η^2^. Cohen’s d was used to measure the magnitude of differences between two specific groups, providing insight into the practical significance of the results. Meanwhile, η^2^ was calculated to quantify the proportion of variance in the dependent variable that could be attributed to the independent variable across multiple groups. This dual approach allowed the understanding of our findings’ statistical and practical relevance.

Interpretation of Cohen’s d:

- Small effect: 0.2 ≤ d < 0.5;
- Medium effect: 0.5 ≤ d < 0.8;
- Large effect: d ≥ 0.8.

Interpretation of η^2^:

- Small effect: 0.01 ≤ η^2^ < 0.060;
- Medium effect: 0.06 ≤ η^2^ < 0.140;
- Large effect: η^2^ ≥ 0.14.

For more details about statistical approaches (Supplementary statistical information—Cohen’s d and η^2^)

Statistical significance was set at *p* < 0.05, and we considered a significance level of *p* < 0.05 as statistically significant, denoting relevance as follows: * *p* < 0.05; ** *p* < 0.01; *** *p* < 0.0015.

## 5. Conclusions

The findings suggest that Doxorubicin enhances sEV production from breast cancer cells. Although this is an in vitro study and not a clinical one, our findings highlight the potential of sEVs derived from cancer cells as a promising approach for diagnosing and prognosing cardiovascular disease in cancer patients, both with and without Doxo treatment. The distinct proinflammatory cytokine profile of these sEVs, marked by elevated TNF-α, IL-6, IL-8, IL-1β, and MCP-1, highlights its role in cardiotoxicity. Monitoring sEV levels and their cytokine content could enhance the safety and effectiveness of Doxo-containing cancer treatments, reducing patients’ risk of cardiac dysfunction and heart failure. Future research should investigate the molecular mechanisms behind sEV-mediated cardiovascular toxicity and assess the feasibility of using these approaches as non-invasive diagnostic tools in clinical practice.

## Data Availability

Data are contained within the article and supplementary materials.

## Supplementary Materials

The following supporting information can be downloaded at: https://www.mdpi.com/article/10.3390/ijms26030945/s1.
