# Supplementary material for: Small Extracellular Vesicles from Breast Cancer Cells Induce Cardiotoxicity
Source: Int J Mol Sci. 2025 Jan 23;26(3):945. doi: 10.3390/ijms26030945 (PMC11816698; doi:10.3390/ijms26030945)
Supplement: Supplementary file 1 [file ijms-26-00945-s001.zip › Supplementary files/Supplementary statistical information.pdf]

This is a supporting material regarding the statistical calculations performed in the manuscript "Small Extracellular Vesicles from Breast Cancer Cells Induce Cardiotoxicity", regarding Cohen's d and eta squared.

ANOVA was the primary method for comparing group differences. Specifically, ANOVA was chosen for its robustness in handling multiple group comparisons.

Additionally, we elaborated on the calculation of effect sizes, including Cohen's d and η^2. Cohen's d was used to measure the magnitude of differences between two specific groups, providing insight into the practical significance of the results. Meanwhile, η^2 was calculated to quantify the proportion of variance in the dependent variable that could be attributed to the independent variable across multiple groups. This dual approach allowed the understanding of our findings' statistical and practical relevance.

Here's a brief explanation of how **Cohen's d** and **η^2 (eta squared)** are computed:

Cohen's d measures the effect size between two groups, quantifying the difference in their means relative to the pooled standard deviation. It is calculated as:

$$d = \frac{M_1 - M_2}{SD_{pooled}}$$

Where:

- M_1 and M_2 are the means of the two groups
- SD_{pooled} is the pooled standard deviation, calculated as:

$$SD_{pooled} = \sqrt{\frac{(n_1 - 1)SD_1^2 + (n_2 - 1)SD_2^2}{n_1 + n_2 - 2}}$$

- SD_1 and SD_2 are the standard deviations of the two groups.
- n_1 and n_2 are the sample sizes of the two groups.

Interpretation of Cohen's d

- **Small effect**: $0.2 \leq d < 0.5$
- **Medium effect**: $0.5 \leq d < 0.8$
- **Large effect**: $d \geq 0.8$

η^2 (Eta Squared)

Eta squared measures the proportion of total variance in the dependent variable that can be attributed to the independent variable in ANOVA. It is calculated as:

$$\eta^2 = \frac{SS_{between}}{SS_{total}}$$

Where:

- $SS_{between}$ is the sum of squares for the variation between groups.
- SS_{total} is the total sum of squares, which includes both between-group and within-group variations.

Interpretation of η^2

- **Small effect**: $0.01 \le \eta^2 < 0.060$
- **Medium effect**: $0.06 \le \eta^2 < 0.140$
- **Large effect**: $\eta^2 \ge 0.14$

These computations provide a quantitative understanding of the magnitude of observed effects, enhancing the interpretability and robustness of statistical results.
