# Supplementary figures and images for: Small Extracellular Vesicles from Breast Cancer Cells Induce Cardiotoxicity
Source: Int J Mol Sci. 2025 Jan 23;26(3):945. doi: 10.3390/ijms26030945 (PMC11816698; doi:10.3390/ijms26030945)

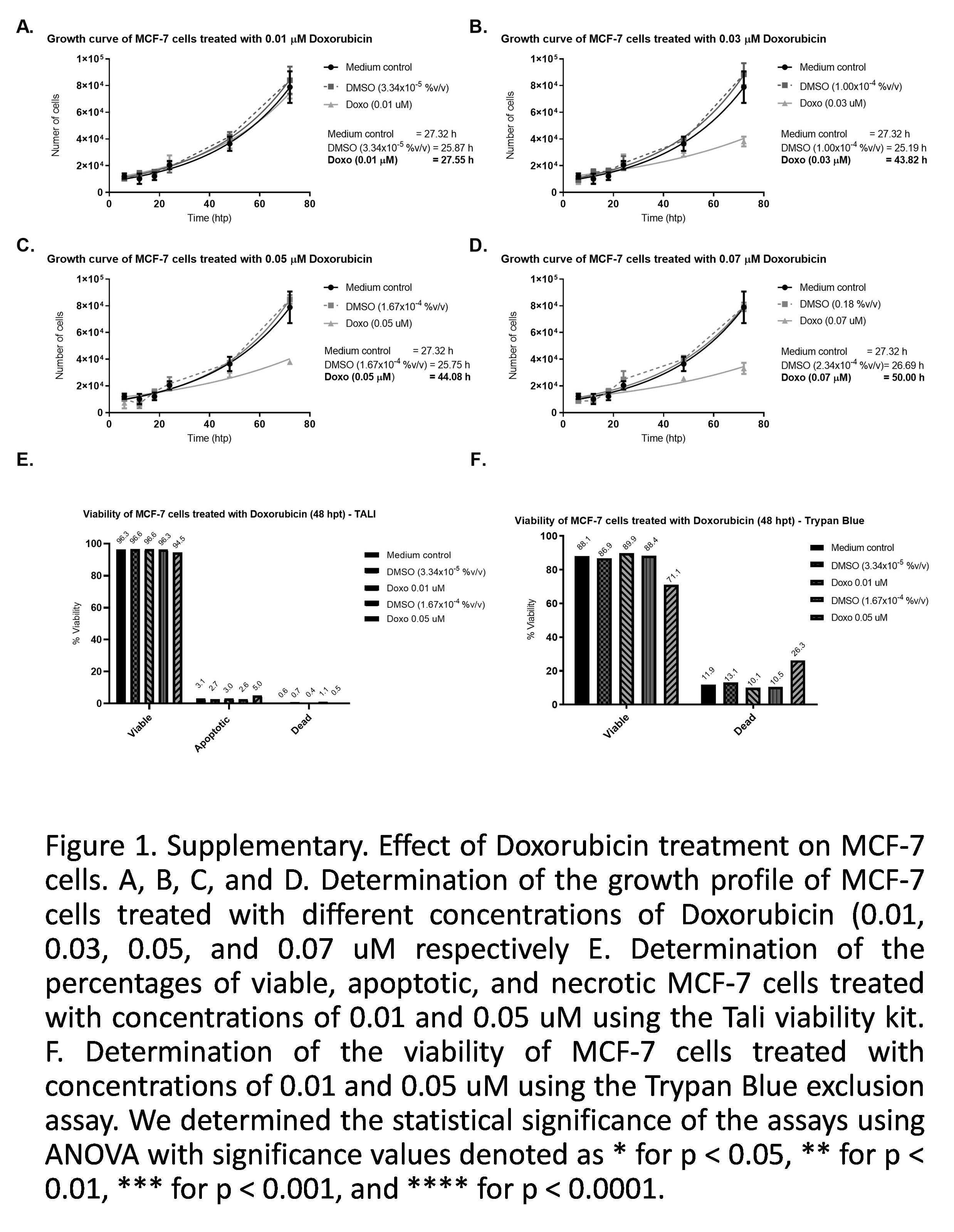

Supplement: Supplementary file 1 [file ijms-26-00945-s001.zip › Supplementary files/Supplementary Figure 1.tif]

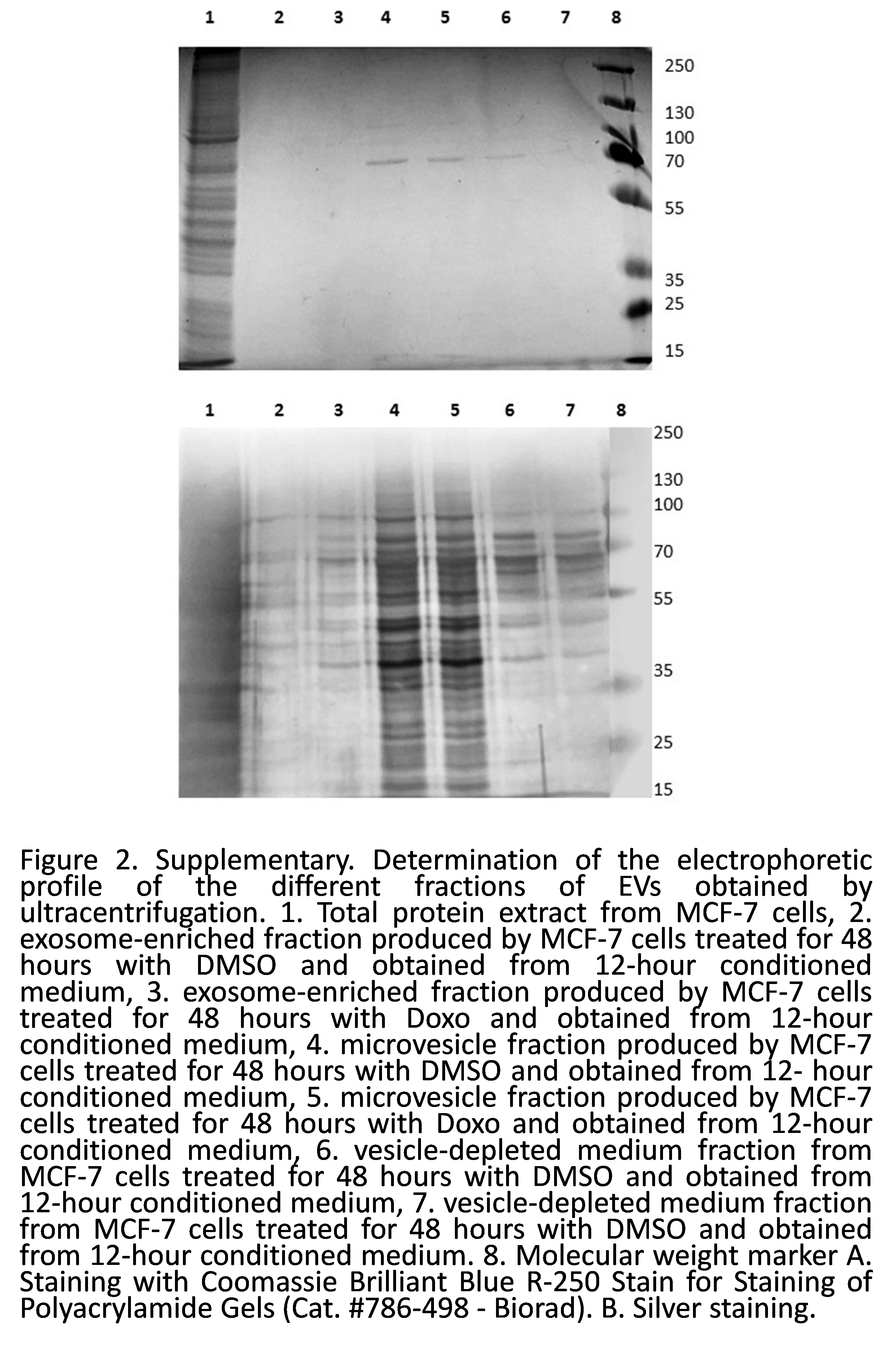

Supplement: Supplementary file 1 [file ijms-26-00945-s001.zip › Supplementary files/Supplementary Figure 2.tif]

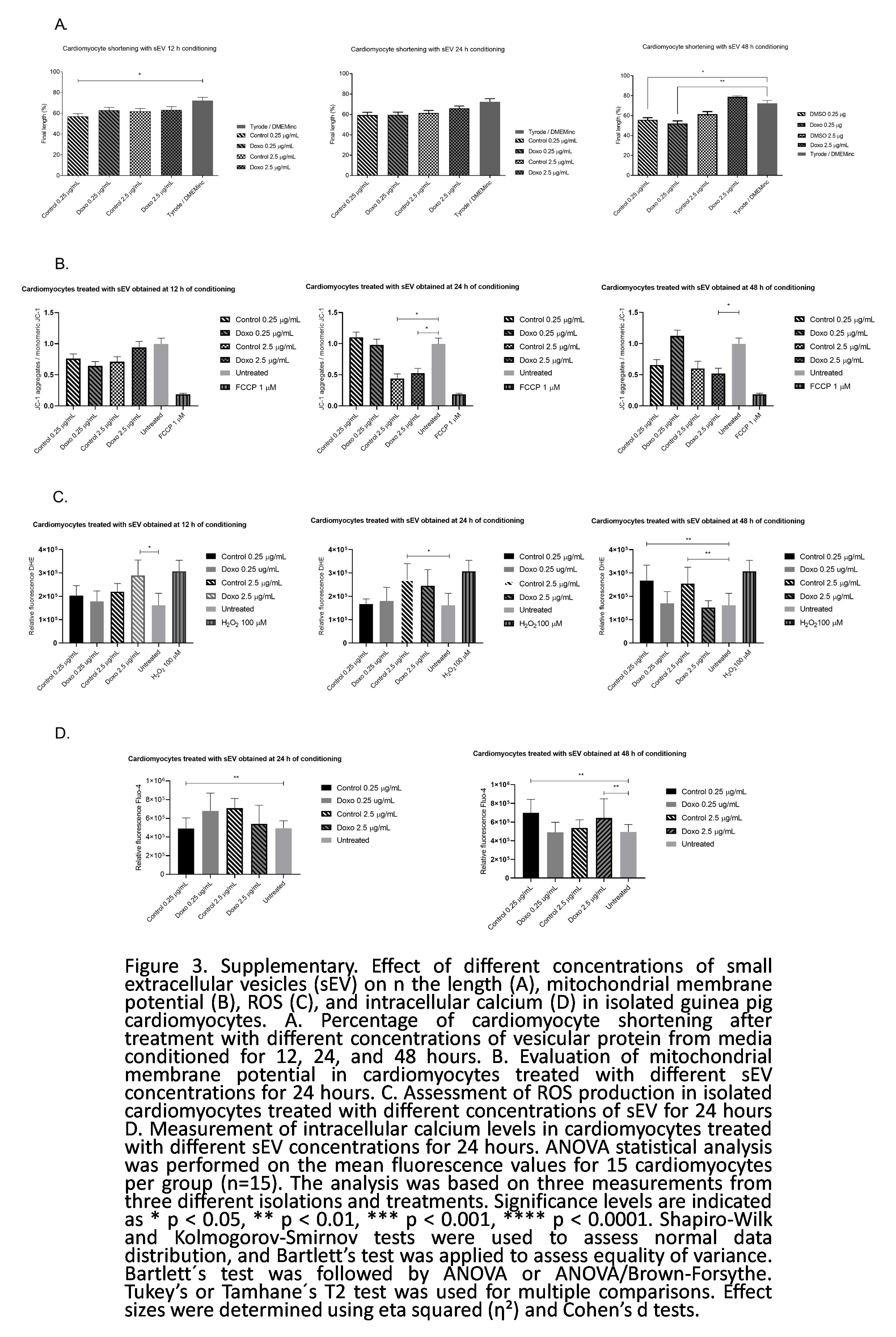

Supplement: Supplementary file 1 [file ijms-26-00945-s001.zip › Supplementary files/Supplementary Figure 3.tif]
